# High-Frequency Dynamics of Ocean pH: A Multi-Ecosystem Comparison

**DOI:** 10.1371/journal.pone.0028983

**Published:** 2011-12-19

**Authors:** Gretchen E. Hofmann, Jennifer E. Smith, Kenneth S. Johnson, Uwe Send, Lisa A. Levin, Fiorenza Micheli, Adina Paytan, Nichole N. Price, Brittany Peterson, Yuichiro Takeshita, Paul G. Matson, Elizabeth Derse Crook, Kristy J. Kroeker, Maria Cristina Gambi, Emily B. Rivest, Christina A. Frieder, Pauline C. Yu, Todd R. Martz

**Affiliations:** 1 Department of Ecology, Evolution and Marine Biology, University of California Santa Barbara, Santa Barbara California, United States of America; 2 Scripps Institution of Oceanography, University of California San Diego, La Jolla, California, United States of America; 3 Monterey Bay Aquarium Research Institute, Moss Landing, California, United States of America; 4 Department of Biology, Stanford University, Hopkins Marine Station, Pacific Grove, California, United States of America; 5 Institute of Marine Sciences, University of California Santa Cruz, Santa Cruz, California, United States of America; 6 Laboratory of Functional and Evolutionary Ecology, Stazione Zoologica Anton Dohrn, Villa Comunale, Naples, Italy; University of California Merced, United States of America

## Abstract

The effect of Ocean Acidification (OA) on marine biota is quasi-predictable at best. While perturbation studies, in the form of incubations under elevated pCO_2_, reveal sensitivities and responses of individual species, one missing link in the OA story results from a chronic lack of pH data specific to a given species' natural habitat. Here, we present a compilation of continuous, high-resolution time series of upper ocean pH, collected using autonomous sensors, over a variety of ecosystems ranging from polar to tropical, open-ocean to coastal, kelp forest to coral reef. These observations reveal a continuum of month-long pH variability with standard deviations from 0.004 to 0.277 and ranges spanning 0.024 to 1.430 pH units. The nature of the observed variability was also highly site-dependent, with characteristic diel, semi-diurnal, and stochastic patterns of varying amplitudes. These biome-specific pH signatures disclose current levels of exposure to both high and low dissolved CO_2_, often demonstrating that resident organisms are already experiencing pH regimes that are not predicted until 2100. Our data provide a first step toward crystallizing the biophysical link between environmental history of pH exposure and physiological resilience of marine organisms to fluctuations in seawater CO_2_. Knowledge of this spatial and temporal variation in seawater chemistry allows us to improve the design of OA experiments: we can test organisms with *a priori* expectations of their tolerance guardrails, based on their natural range of exposure. Such hypothesis-testing will provide a deeper understanding of the effects of OA. Both intuitively simple to understand and powerfully informative, these and similar comparative time series can help guide management efforts to identify areas of marine habitat that can serve as refugia to acidification as well as areas that are particularly vulnerable to future ocean change.

## Introduction

Since the publication of two reports in 2005–2006 [1], [2], the drive to forecast the effects of anthropogenic ocean acidification (OA) on marine ecosystems and their resident calcifying marine organisms has resulted in a growing body of research. Numerous laboratory studies testing the effects of altered seawater chemistry (low pH, altered pCO_2_, and undersaturation states - Ω - for calcium carbonate polymorphs) on biogenic calcification, growth, metabolism, and development have demonstrated a range of responses in marine organisms (for reviews see [3]–[8]). However, the emerging picture of biological consequences of OA – from data gathered largely from laboratory experiments – is not currently matched by equally available environmental data that describe present-day pH exposures or the natural variation in the carbonate system experienced by most marine organisms. Although researchers have documented variability in seawater carbonate chemistry on several occasions in different marine ecosystems (e.g., [9]–[15]), this variation has been under-appreciated in these early stages of OA research.

Recently, a deeper consideration of ecosystem-specific variation in seawater chemistry has emerged (e.g., [16]–[18]), one that is pertinent to the study of biological consequences of OA. Specifically, assessments of environmental heterogeneity present a nuanced complement to current laboratory experiments. The dynamics of specific natural carbonate chemistry on local scales provide critical context because outcomes of experiments on single species are used in meta-analyses to project the overall biological consequences of OA [7], [19], to forecast ecosystem-level outcomes [20], and ultimately to contribute to policy decisions [21] and the management of fisheries [22], [23]. As noted earlier [24], natural variability in pH is seldom considered when effects of ocean acidification are considered. Natural variability may occur at rates much higher than the rate at which carbon dioxide is decreasing ocean pH, about −0.0017 pH/year [25], [26]. This ambient fluctuation in pH may have a large impact on the development of resilience in marine populations, or it may combine with the steady effects of acidification to produce extreme events with large impacts [24]. In either case, understanding the environmental variability in ocean pH is essential.

Although data on the natural variation in the seawater CO_2_ system are emerging, nearly all high-resolution (*e.g.* hourly) time series are based on pCO_2_ sensors, with comparatively few pH time series found in the literature. From a research perspective, the absence of information regarding natural pH dynamics is a critical data gap for the biological and ecological arm of the multidisciplinary investigation of OA. Our ability to understand processes ranging from physiological tolerances to local adaptation is compromised. Specifically, laboratory experiments to test tolerances are often not designed to encompass the actual habitat exposure of the organisms under study, a critical design criterion in organismal physiology that also applies to global change biology [27]–[29]. It is noted that neither pH nor pCO_2_ alone provide the information sufficient to fully constrain the CO_2_ system, and while it is preferred to measure both, the preference for measuring one over the other is evaluated on a case-by-case basis and is often dictated by the equipment available.

In this light, data that reveal present-day pH dynamics in marine environments and therefore ground pH levels in CO_2_ perturbation experiments in an environmental context are valuable to the OA research community in two major ways. First, estimates of organismal resilience are greatly facilitated. Empiricists can contextualize lab experiments with actual environmental data, thereby improving them. Notably, the majority of manipulative laboratory experiments in OA research (including our own) have been parameterized using pCO_2_ levels as per the IPCC emission scenario predictions [30]. One consequence of this practice is that organisms are potentially tested outside of the current exposure across their biogeographic range, and tolerances are not bracketed appropriately. This situation may not be a lethal issue (*i.e.* negating all past observations in experiments where environmental context was not known); however, the lack of information about the ‘pH seascape’ may be translated through these organismal experiments in a manner that clouds the perspective of vulnerability of marine ecosystems. For example, recent data on the heterogeneity of pH in coastal waters of the Northeastern Pacific [31], [32] that are characterized by episodic upwelling has caused biologists to re-examine the physiological tolerances of organisms that live there. Specifically, resident calcifying marine invertebrates and algae are acclimatized to existing spatial and temporal heterogeneity [17], [18], and further, populations are likely adapted to local to regional differences in upwelling patterns [33].

Secondly, in addition to improving laboratory experiments, data regarding the nature of the pH seascape also facilitate hypothesis-generating science. Specifically, heterogeneity in the environment with regard to pH and pCO_2_ exposure may result in populations that are acclimatized to variable pH or extremes in pH. Although this process has been highlighted in thermal biology of marine invertebrates [34], such insight is not available with regard to gradients of seawater chemistry that occur on biogeographic scales. With that said, recent field studies have demonstrated that natural variation in seawater chemistry does influence organismal abundance and distribution [16], [35], [36]. With our newfound access to pH time series data, we can begin to explore the biophysical link between environmental seawater chemistry and resilience to baseline shifts in pH regimes, to identify at-risk populations as well as tolerant ones. Additionally, the use of sensors in the field can identify hidden patterns in the CO_2_ system, revealing areas that are refugia to acidification or carbonate undersaturation; such knowledge could enable protection, management, and remediation of critical marine habitats and populations in the future.

The recent development of sensors for *in situ* measurements of seawater pH [37], [38] has resulted in the ability to record pH more readily in the field in a manner that can support biological and ecological research. Since 2009, the Martz lab (SIO) has constructed 52 “SeaFET” pH sensors for 13 different collaborators (see http://martzlab.ucsd.edu) working in a broad range of settings. Using subsamples of data from many of these sensors, here we examine signatures of pH heterogeneity, presenting time series snapshots of sea-surface pH (upper 10 m) at 15 locations, spanning various *overlapping* habitat classifications including polar, temperate, tropical, open ocean, coastal, upwelling, estuarine, kelp forest, coral reef, pelagic, benthic, and extreme. Naturally, at many sites, multiple habitat classifications will apply. Characteristic patterns observed in the 30-day snapshots provide biome-specific pH signatures. This comparative dataset highlights the heterogeneity of present-day pH among marine ecosystems and underscores that contemporary marine organisms are currently exposed to different pH regimes in seawater that are not predicted until 2100.

## Results

**Figure 1:**
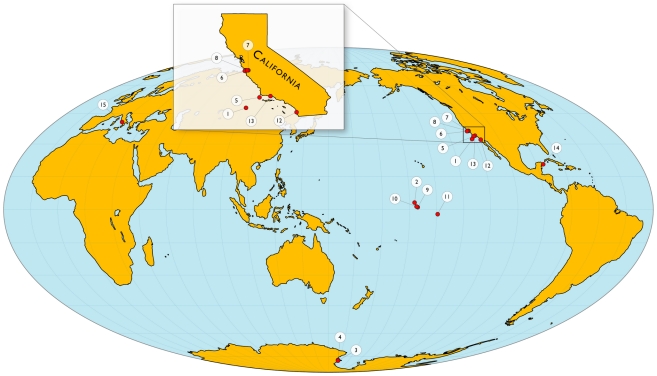
Map of pH sensor (SeaFET) deployment locations. See Table 1 for details regarding deployment locations.

**Figure 2:**
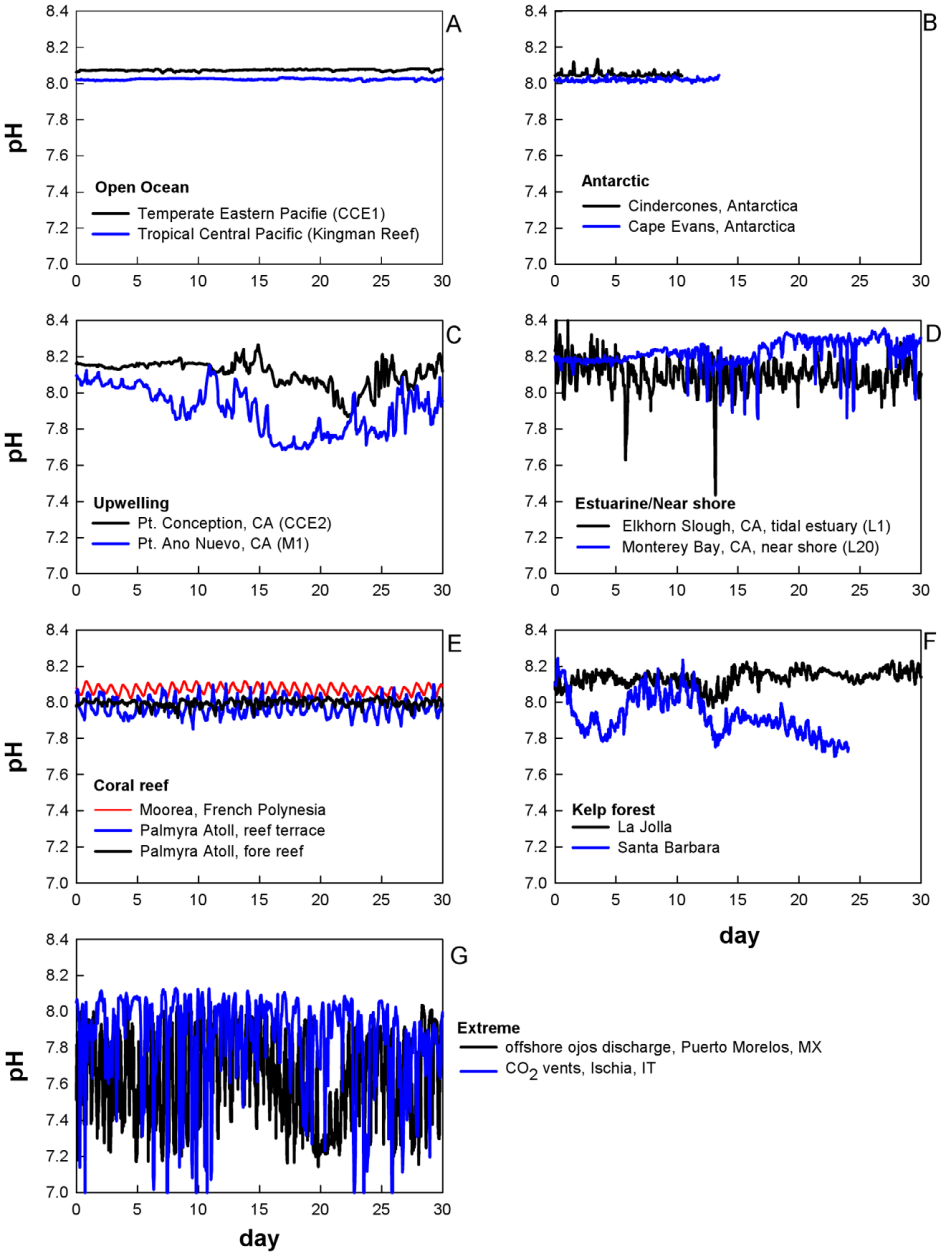
pH dynamics at 15 locations worldwide in 0–15 m water depth. All panels are plotted on the same vertical range of pH (total hydrogen ion scale). The ordinate axis was arbitrarily selected to encompass a 30-day period during each sensor deployment representative of each site during the deployment season. See Table 1 for details regarding sensor deployment.

**Figure 3:**
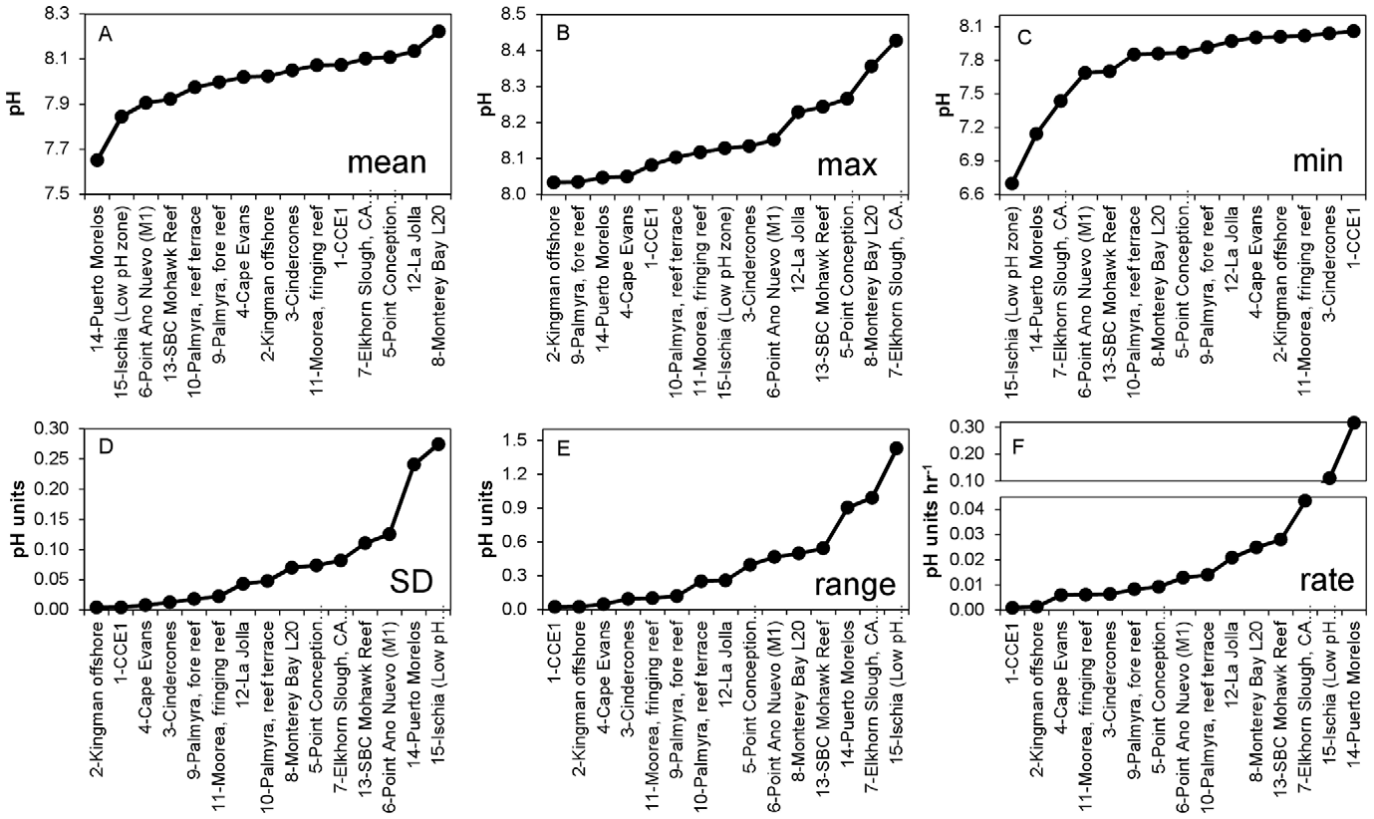
Metrics of short-term pH variability at 15 locations worldwide, ranked by ascending values. Mean = geometric mean; Max = maximum value recorded; Min = minimum value recorded; SD = standard deviation; Range = Max - Min; Rate = mean of the absolute rate of change between adjacent data points.

Overall, the patterns of pH recorded at each of the 15 deployment sites (shown in Figure 1, Table 1) were strikingly different. Figure 2 presents the temporal pattern of pH variation at each of these sites, and, for the sake of comparison, these are presented as 30-day time series “snapshots.” Note that all deployments generated >30 days of data except for sensors 3, 4, and 13, where the sensors were deliberately removed due to time constraints at the study sites. Though the patterns observed among the various marine ecosystems are driven by a variety of oceanographic forcing such as temperature, mixing, and biological activity, we do not provide a separate analysis of controlling factors on pH at each location. Each time series was accompanied by a different set of ancillary data, some rich with several co-located sensors, others devoid of co-located sensors. Given these differences in data collection across sites, here we focus on the comparative pH sensor data as a means to highlight observed pH variability and ecosystem-level differences between sites. For purposes of comparison, the metrics of variability presented here are pH minima, maxima, range, standard deviation, and rate of change (see Table 2). The rate presented in Table 2 and Figure 3 represents a mean instantaneous rate of change in pH hr^−1^, where a rate was calculated for each discrete time step as the absolute value of pH difference divided by the length of time between two adjacent data points.

**Table 1:** Summary of pH sensor deployment data shown in Figure 1.

Category	Sensor-Site	Latitude	Longitude	DD^1^	WD^2^	t_o_^3^	PI
Open Ocean(p)	1-CCE1	33.5 N	122.5 W	2	4000	6-Mar-2011	Send
Reef(b)	2-Kingman offshore	6.43961 N	162.3949 W	10	10	27-Apr-2010	Smith
Polar(b)	3-Cindercones	77.8000 S	166.6712 E	15	16	12-Oct-2010	Hofmann
Polar(b)	4-Cape Evans	77.6343 S	166.4484 E	15	16	2-Nov-2010	Hofmann
Upwelling(p)	5-Point Conception (CCE2)	34.32 N	120.80 W	2	770	27-Mar-2011	Send
Upwelling(p)	6-Point Ano Nuevo (M1)	36.8 N	122 W	2	800	15-Apr-2010	Johnson
Tidal Estuary(p)	7-Elkhorn Slough, CA (L1)	36.8125 N	121.7748 W	1	8	15-Sep-2008	Johnson
Near Shore(p)	8-Monterey Bay L20	36.8135 N	121.8290 W	1	19	1-Aug-2010	Johnson
Reef(b)	9-Palmyra, fore reef	5.86614 N	162.1172 W	10	10	20-Apr-2010	Smith
Reef(b)	10-Palmyra, reef terrace	5.884 N	162.1218 W	5	5	19-Apr-2010	Smith
Reef(b)	11-Moorea, fringing reef	17.4803 S	149.7989 W	10	11	10-Feb-2011	Hofmann
Kelp(p)	12-La Jolla	32.80853 N	117.2890 W	7	20	28-Jul-2010	Levin
Kelp(b)	13-SBC Mohawk Reef	34.3943 N	119.73 W	8	9	24-Jul-2010	Hofmann
Extreme(b)	14-Puerto Morelos	20 N	86.5 W	5	5	27-Aug-2010	Paytan
Extreme(b)	15-Ischia (South zone)	40.7303 N	13.9636 E	1	3	10-May-2010	Micheli

p = pelagic.

b = benthic.

1-Deployment Depth in meters.

2-Water Depth in meters.

3-Starting time of the 30-day window shown in Figure 1.

**Table 2:** Summary data for pH temporal profiles collected at 15 sensor locations.

Site	Mean	Max	Min	SD	Range	Rate^1^
CCE-1	8.074	8.082	8.059	0.004	0.024	0.001
Kingman Reef	8.023	8.034	8.009	0.004	0.025	0.001
Cindercones	8.050	8.134	8.039	0.013	0.096	0.006
Cape Evans	8.020	8.050	8.002	0.008	0.047	0.006
Pt. Conception (CCE2)	8.108	8.266	7.869	0.074	0.397	0.009
Pt. Ano Nuevo	7.905	8.152	7.685	0.126	0.467	0.013
Elkhorn Slough (L1)	8.101	8.427	7.435	0.082	0.992	0.043
Monterey Bay (M1)	8.222	8.356	7.857	0.070	0.499	0.025
Palmyra, fore reef	7.997	8.035	7.915	0.018	0.121	0.008
Palmyra, reef terrace	7.974	8.104	7.851	0.048	0.253	0.014
Moorea, fringing reef	8.072	8.118	8.017	0.022	0.101	0.006
La Jolla	8.134	8.229	7.970	0.043	0.259	0.021
SBC Mohawk Reef	7.922	8.244	7.700	0.111	0.544	0.028
Puerto Morelos	7.651	8.048	7.143	0.241	0.905	0.317
Ischia (South zone)	7.845	8.129	6.699	0.274	1.430	0.110

1-calculated as mean(abs(pH_t2_−pH_t1_)/(t_2_−t_1_)).

In terms of general patterns amongst the comparative datasets, the open ocean sites (CCE1 and Kingman Reef) and the Antarctic sites (Cape Evans and Cindercones) displayed the least variation in pH over the 30-day deployment period. For example, pH range fluctuated between 0.024 to 0.096 at CCE1, Kingman Reef, Cape Evans, and Cindercones (Figure 2A, B and Table 2). In distinct contrast to the stability of the open ocean and Antarctic sites, sensors at the other five site classifications (upwelling, estuarine/near-shore, coral reef, kelp forest, and extreme) captured much greater variability (pH fluctuations ranging between 0.121 to 1.430) and may provide insight towards ecosystem-specific patterns. The sites in upwelling regions (Pt. Conception and Pt. Ano Nuevo, Figure 2C), the two locations in Monterey Bay, CA (Figure 2D), and the kelp forest sites (La Jolla and Santa Barbara Mohawk Reef, Figure 2F) all exhibited large fluctuations in pH conditions (pH changes>0.25). Additionally, at these 6 sites, pH oscillated in semi-diurnal patterns, the most apparent at the estuarine sites. The pH recorded in coral reef ecosystems exhibited a distinct diel pattern characterized by relatively consistent, moderate fluctuations (0.1<pH change<0.25; Figure 2E). At the Palmyra fore reef site, pH maxima occurred in the early evening (∼5:00 pm), and pH minima were recorded immediately pre-dawn (∼6:30 am). On a fringing reef site in Moorea, French Polynesia, a similar diel pattern was observed, with pH maxima occurring shortly after sunset (∼7:30 pm) and pH minima several hours after dawn (∼10:00 am). Finally, the greatest transitions in pH over time were observed at locations termed our “Extreme” sites - a CO_2_ venting site in Italy (site S2 in ref. [36]) and a submarine spring site in Mexico. For these sites, the patterns were extremely variable and lacked a detectable periodicity (Figure 2G).

The sites examined in this study do not comprehensively represent pH variability in coastal ecosystems, partly because we focused on surface epipelagic and shallow benthic pH variability. Many organisms that may be impacted by pH variability and ocean acidification reside at intermediate (>10 m) to abyssal depths. Notable regimes missing from Figure 2 include seasonally stratified open ocean locations that exhibit intense spring blooms; the equatorial upwelling zone; other temperate (and highly productive) Eastern Continental Boundary upwelling areas; subsurface oxygen minimum zones and seasonal dead zones; and a wide variety of unique estuarine, salt marsh, and tide pool environments. Spring bloom locations exhibit a marked increase in diel pCO_2_ variability during the peak bloom with a coincident drawdown similar in magnitude but opposite in sign to the upwelling signals shown in Figure 2 [39]. Equatorial upwelling locations undergo significant stochastic variability, as observed by pCO_2_ sensors in the TAO array (data viewable at http://www.pmel.noaa.gov/). Intertidal vegetated and tide pool habitats may exhibit major pH fluctuations due to macrophyte or animal respiratory cycles [15], while CO_2_ production in oxygen minimum zones can reduce pH to a limit of about 7.4 [40].

Due to local temperature differences, variable total alkalinity, and seasonal differences between deployment dates at each site, a comparison of average pH across the datasets would be somewhat misleading. However, some information can be gleaned from an examination of the averages: the overall binned average of all 15 mean values in Table 1 is 8.02±0.1. This pH value is generally in agreement with the global open ocean mean for 2010 of 8.07, a value generated by combining climatology data for temperature, salinity, phosphate, silicate [41]–[43], total alkalinity [44], and pCO_2_ [45] for the year 2000, corrected to 2010 using the average global rise of 1.5 µatm pCO_2_ yr^−1^. Rather than make a point-by-point comparison of the mean pH of each dataset, we focus instead on the differences in observed variability amongst the sites. For this analysis, summary statistics of the comparative datasets were ranked in order to examine the range of variability across all 15 sites (Fig. 3).

## Discussion

Collected by 15 individual SeaFET sensors in seven types of marine habitats, data presented here highlight natural variability in seawater pH. Based on Figure 3, it is evident that regions of the ocean exhibit a continuum of pH variability. At sites in the open ocean (CCE-1), Antarctica, and Kingman reef (a coastal region in the permanently stratified open Pacific Ocean with very low residence times, and thus representative of the surrounding open ocean water), pH was very stable (SD<0.01 pH over 30 days). Elsewhere, pH was highly variable across a range of ecosystems where sensors were deployed. The salient conclusions from this comparative dataset are two-fold: (1) most non-open ocean sites are indeed characterized by natural variation in seawater chemistry that can now be revealed through continuous monitoring by autonomous instrumentation, and (2) in some cases, seawater in these sites reaches extremes in pH, sometimes daily, that are often considered to only occur in open ocean systems well into the future [46]. Admittedly, pH is only part of the story with regard to the biological impacts of OA on marine organisms. However, continuous long-term observations provided by sensors such as the SeaFET are a great first step in elucidating the biophysical link between natural variation and physiological capacity in resident marine organisms.

In the end, knowledge of spatial and temporal variation in seawater chemistry is a critical resource for biological research, for aquaculture, and for management efforts. From a biological perspective, the evolutionary history of the resident organisms will greatly influence the adaptation potential of organisms in marine populations. Thus, present-day natural variation will likely shape capacity for adaptation of resident organisms, influencing the resilience of critical marine ecosystems to future anthropogenic acidification. Below we discuss the comparative SeaFET-collected data and, where applicable, the biological consequences of the temporal heterogeneity that we found in each of the marine ecosystems where sensors were deployed.

**Figure 4:**
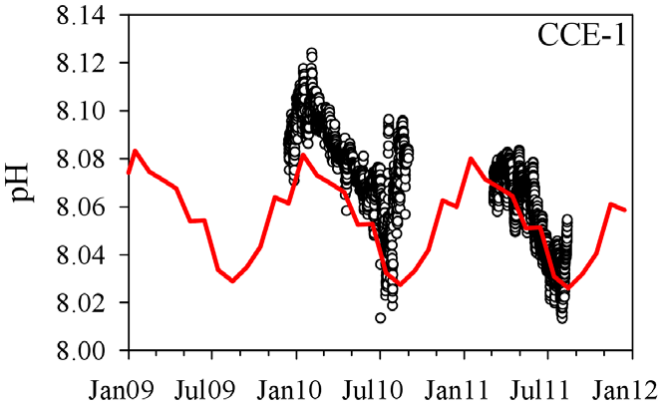
Comparison between sensor data (symbols) and the pH climatology (line) near CCE-1.

As the most stable area, the open ocean behaves in a predictable way and generally adheres to global models attempting to predict future CO_2_ conditions based on equilibration of the surface ocean with a given atmospheric pCO_2_ (e.g. [47]). This can be shown with longer-term pH records obtained with SeaFET sensors, which are available at the CCE-1 mooring (Fig. 4). The ambient pH values for this open ocean location can be predicted to better than ±0.02 from the CO_2_-corrected climatology mentioned above; pH has dropped by about 0.015 units since 2000. At CCE-1, the annual carbonate cycle followed the sea surface temperature cycle, and pH was driven mostly by changes in the temperature dependence of CO_2_ system thermodynamics (Figure 4). SeaFET observations at CCE-1 agree with the climatology to +0.017±0.014 pH units, with episodic excursions from the climatology but a general return to the climatological mean. Although the annual cycle in the open ocean is somewhat predictable, it is notable that even at these seemingly stable locations, climatology-based forecasts consistently underestimate natural variability. Our observations confirm an annual mean variability in pH at CCE-1 of nearly 0.1, suggest an inter-annual variability of ∼0.02 pH, and capture episodic changes that deviate from the climatology (Figure 4). Similar underestimates of CO_2_ variability were observed at nine other open ocean locations, where the Takahashi pCO_2_ climatology overlaps PMEL moorings with pCO_2_ sensors (not shown). Thus, on both a monthly (Fig. 2) and annual scale (Fig. 4), even the most stable open ocean sites see pH changes many times larger than the annual rate of acidification. This natural variability has prompted the suggestion that “an appropriate null hypothesis may be, until evidence is obtained to the contrary, that major biogeochemical processes in the oceans other than calcification will not be fundamentally different under future higher CO_2_/lower pH conditions” [24].

Similarly, the sensors deployed on the benthos in the Antarctic (Cindercones and Cape Evans, Figure 2B) recorded relatively stable pH conditions when compared to other sites in the study. Very few data exist for the Southern Ocean; however, open-water areas in this region experience a strong seasonal shift in seawater pH (∼0.3–0.5 units) between austral summer and winter [48], [49] due to a decline in photosynthesis during winter and a disequilibrium of air-sea CO_2_ exchange due to annual surface sea ice and deep water entrainment [50]. Given the timing of deployment of our sensor in McMurdo Sound (austral spring: October–November), the sensor did not capture the change in seawater chemistry that might have occurred in the austral winter [49]. In general, due to sea ice conditions, observations from the Southern Ocean are limited, with water chemistry data falling into two categories: (1) discrete sampling events during oceanographic cruises (e.g. US Joint Global Ocean Flux Study, http://www1.whoi.edu/) and (2) single-point measurements from locations under sea ice [49], [51], [52]. Biologically speaking, the Southern Ocean is a region expected to experience acidification and undersaturated conditions earlier in time than other parts of the ocean [47], and calcifying Antarctic organisms are thought to be quite vulnerable to anthropogenic OA given the already challenging saturation states that are characteristic of cold polar waters [53]–[56]. Short-term CO_2_ perturbation experiments have shown that Antarctic calcifying marine invertebrates are sensitive to decreased saturation states [51], [57], although the number of species-level studies and community-level studies are very limited. The Western Antarctic Peninsula and the sub-Antarctic islands will experience pronounced increases in temperature [54] and could consequently undergo more variation and/or undersaturation given the increased potential for biological activity. Importantly, depending on the patterns of seasonally-dependent saturation state that will be revealed with improved observations [58], Antarctic organisms may experience more variation than might be expected, a situation that will influence their resilience to future acidification.

Three other types of study sites – the coastal upwelling, kelp forest and estuarine/near-shore sites – all exhibited variability due to a combination of mixing, tidal excursions, biological activity, and variable residence time (Fig. 2). Although these sites are all united by fairly obvious heterogeneity in pH, organisms living in these areas encounter unique complexities in seawater chemistry that will influence their physiological response, resilience, and potential for adaptation.

Typically, estuarine environments have riverine input that naturally creates very low saturation states [59]–[61]. Seawater chemistry conditions in these areas often shift dramatically, challenging biogenic calcification by resident organisms. Additionally, these species must also tolerate abiotic factors that interact with pH, such as temperature [62]. Two sensors in the Monterey Bay region, L1 (at the mouth of Elkhorn Slough) and L20 (∼2 km seaward and north of L1), recorded rapid changes in pH. However, as opposed to riverine input, the low pH fluctuations observed here are likely due to isopycnal shoaling or low CO_2_ water that is pulsing up to the near shore on internal tides. These locations may also experience high river run-off in the rainy season, but such conditions were not reflected in the time series shown in Fig. 2.

Organisms living in upwelling regions may be acclimatized and adapted to extremes in seawater chemistry; here, deep CO_2_-enriched waters reach the surface and may shoal onto the benthos on the continental shelf [31], [32]. Data collected from our upwelling sites support the patterns found by cruise-based investigations; pH fluctuations were often sharp, and large transitions of up to ∼0.35 pH units occurred over the course of days (Fig. 2). Laboratory studies on calcifying marine invertebrates living in upwelling regions suggest that these organisms maintain function under such stochastic conditions. However, overall performance may be reduced, suggesting that these species are indeed threatened by future acidification [17], [18], [63].

For kelp forests, although there is less influence from riverine inputs, pH variation is quite dynamic at these sites in the coastal California region (Fig 2; [18]). Patterns here are likely driven by fluctuations in coastal upwelling, biological activity, currents, internal tides, seasonally shoaling isopleths, as well as the size of the kelp forest, which may influence residence times via reduced flow. Kelps may respond positively to increased availability of CO_2_ and HCO_3_^−^, which may allow for reduced metabolic costs and increased productivity [64]. Increased kelp production may elevate pH within the forest during periods of photosynthesis, causing wider daily fluctuations in pH, though this is speculative at this time. As a result, kelp forests, particularly those of surface canopy forming species such as *Macrocystis pyrifera*, may contain a greater level of spatial heterogeneity in terms of the pH environment; vertical gradients in pH may form due to enhanced levels of photosynthesis at shallower depths. Such gradients may increase the risk of low pH exposure for benthic species while buffering those found within the surface canopy. Kelp forests provide habitat to a rich diversity of organisms from a wide range of calcifying and non-calcifying taxa [65]. As with organisms from the other coastal locations (estuarine and upwelling), the biota living within kelp forest environments are most likely acclimatized to this degree of natural variation. However, continued declines in oxygenation and shoaling of hypoxic boundaries observed in recent decades in the southern California bight [66], [67] are likely accompanied by a reduction in pH and saturation state. Thus, pH exposure regimes for the coastal California region's kelp forest biota may be changing over relatively short time scales. Over longer temporal scales as pH and carbonate saturation levels decrease, the relative abundances of these species may change, with community shifts favoring non-calcified species, as exemplified by long-term studies in intertidal communities by Wootton et al. [15].

For all the marine habitats described above, one very important consideration is that the extreme range of environmental variability does not necessarily translate to extreme resistance to future OA. Instead, such a range of variation may mean that the organisms resident in tidal, estuarine, and upwelling regions are already operating at the limits of their physiological tolerances (*a la* the classic tolerance windows of Fox – see [68]). Thus, future acidification, whether it be atmospheric or from other sources, may drive the physiology of these organisms closer to the edges of their tolerance windows. When environmental change is layered upon their present-day range of environmental exposures, they may thereby be pushed to the “guardrails” of their tolerance [20], [68].

In contrast to more stochastic changes in pH that were observed in some sites, our coral reef locations displayed a strikingly consistent pattern of diel fluctuations over the 30-day recording period. Similar short-term pH time series with lower daily resolution [69], [70] have reported regular diel pH fluctuation correlated to changes in total alkalinity and oxygen levels. These environmental patterns of pH suggest that reef organisms may be acclimatized to consistent but moderate changes in the carbonate system. Coral reefs have been at the center of research regarding the effects of OA on marine ecosystems [71]–[73]. Along with the calcification biology of the dominant scleractinian corals and coralline algae, the biodiversity on coral reefs includes many other calcifying species that will likely be affected [74]–[77]. Across the existing datasets in tropical reef ecosystems, the biological response of calcifying species to variation in seawater chemistry is complex (see [78]) –all corals or calcifying algal species will not respond similarly, in part because these calcifying reef-builders are photo-autotrophs (or mixotrophs), with algal symbionts that complicate the physiological response of the animal to changes in seawater chemistry.

Finally, the “Extreme” sites in our comparative dataset are of interest in that the low pH levels observed here represent a natural analogue to OA conditions in the future, demonstrating how the abundance and distribution of calcifying benthic organisms, as well as multi-species assemblages, can vary as a function of seawater chemistry [16], [35], [36], [79]. The variability in seawater pH was higher at both the groundwater springs off the coast of Mexico and the natural CO_2_ vents off the coast of Italy than at any of the other sensor locations. Offshore of Puerto Morelos, Mexico (and at other sites along the Mesoamerican Reef), natural low-saturation (Ω∼0.5, pH 6.70–7.30, due to non-ventilated, high CO_2_, high alkalinity groundwater) submarine springs have been discharging for millennia. Here, variability in pH is due to long-term respiration driving a low ratio of alkalinity to dissolved inorganic carbon in effluent ground water. These sites provide insight into potential long-term responses of coral backreef ecosystems to low saturation conditions [79]. Unlike Puerto Morelos, the variability of pH at volcanic CO_2_ vents at Ischia, Italy is almost purely abiotically derived, due entirely to CO_2_ venting and subsequent mixing. This site in the Mediterranean Sea hosts a benthic assemblage that reflects the impacts of OA on rocky reef communities [16], [36].

Overall, the ‘extreme’ systems provide an opportunity to examine how variability in pH and extreme events (*sensu* [80]) affects ecological processes. Knowledge of this biophysical link is essential for forecasting ecological responses to acidification in ecosystems with sharp fluctuations in pH, such as upwelling or estuarine environments. Despite reductions in species richness, several calcifying organisms are found in low pH conditions close to the vents [16] and the springs [79]. The persistence of calcifying organisms at these extreme sites, where mean pH values are comparable to those that have reduced organism performance in laboratory experiments (i.e., pH_T_ 7.8; reviewed in [16]), suggest that long exposures to such variability in pH, versus a consistently low-pH environment, could play an important role in regulating organism performance. Variability in pH could potentially promote acclimatization or adaptation to acidification through repeated exposure to low pH conditions [24]; alternatively, transient exposures to high pH conditions could buffer the effects of acidification by relieving physiological stress. Thus, the ecological patterns coupled with the high fluctuations in pH at the extreme sites highlight the need to consider carbonate chemistry variability in experiments and models aimed at understanding the impacts of acidification.

Examination of Figure 3 indicates that no single simple statistical metric is sufficient to characterize the pH of an ecosystem. Not surprisingly, there is considerable overlap between panels D, E and F, confirming that sites with the greatest range and variance will exhibit the greatest instantaneous rates of change. While these simple metrics are easily understood and therefore helpful in a descriptive sense, the fact that observed pH may not exhibit a normal distribution should serve as a reminder that each system is unique and complex. Depending on the mechanism (*e.g.* mixing vs. biological) driving the observed change, the metric used to characterize the system will hold a different meaning. For example, during the periods compared here, the sensor in Monterey Bay at M1 (Fig. 2C) has a higher SD and range than the sensor located in the La Jolla Kelp forest (Fig. 2F), yet the average instantaneous rate of change in the La Jolla Kelp forest is higher than at M1 (Fig. 3D–F).

In summary, together, these pH time series create a compelling argument for the collection of more continuous data of this kind. Specifically, these data represent a critical step in understanding the consequences of ocean change: the linkage of present-day pH exposures to organismal tolerance and how this translates into ecological change in marine ecosystems [27], [81]. Long-term datasets exist, but many are in open-ocean locations (HOTS, BATS, ESTOC) and do not capture environmental variation in the coastal marine habitats that are of such critical ecological and economic value [20]. Additionally, they often do not capture changes in pH at physiologically relevant timescales since they are limited by ship-board sampling frequencies. The processes that combine to drive changes in seawater chemistry are complex; water chemistry at any location in the ocean is a result of air-sea exchange, land-water interactions, and the physical, chemical, and biological processes occurring in the water column and on the benthos. In the shallow coastal ecosystems measured here, the combination of background oceanography, resident biological processes, and residence time of the water are likely driving the daily variability or lack thereof in seawater pH. All of these processes can contribute to the mean, minimum, maximum, and diurnal or seasonal variability occurring within and among ecosystems or habitats. At this juncture, it is not clear what aspect of this variability is most biologically significant (*e.g.* minimum pH, maximum pH, hours spent below the yearly mean low pH); however, investigations in thermal biology have begun to tease apart the parameters that are relevant to organismal physiology [27].

As a final note, we do concede that, like pCO_2_, pH may not tell the whole story. It may in fact be saturation state and not pH that is the main driver of the mechanistic and physiological impact of OA, at least for calcifying organisms. While our comparative data set provides a unique look into natural pH variability, the overall picture of variability in the carbonate system will remain incomplete until we are able to fully characterize the CO_2_ system with additional sensors for dissolved inorganic carbon and alkalinity. There is also a need to monitor other hydrographic variables in addition to the carbonate system. Modern and paleo OA events are accompanied by shifts in temperature, stratification, and dissolved oxygen [82]. The impacts of OA can depend on values of interacting stressors such as temperature and oxygen, and pH may in turn alter tolerance to these stressors, with major consequences for organism function [83]. However, at present, the use of autonomous sensors can greatly improve our perspectives of how future acidification might influence species physiology, fitness, and interactions in marine ecosystems.

## Materials and Methods

All necessary permits were obtained for the described field studies. Permits were issued for sensor deployments by: Moss Landing Harbor District (L01); Monterey Bay National Marine Sanctuary (L20 and M1); Délégation à la Recherche, Territorial Government of French Polynesia (Moorea Coral Reef LTER); US Fish and Wildlife (Palmyra Atoll National Wildlife Refuge). For all other sites in this study, no specific permits were required for sensor deployments.

The pH sensors used in this study are based on a modified version of the Honeywell DuraFET®, an ion sensitive field effect transistor (ISFET), with an integrated data logger and power supply [37]. We refer to these autonomous versions of the DuraFET as “SeaFET”. Data presented in Figure 2 were recorded by several different generations of SeaFET, with the most notable difference being that sensors 1, 5, 8, 12, and 14 were operated inside of a flow manifold that was flushed with a submersible pump (Sea-Bird SBE5) before each measurement while the other ten sensors were protected by a passively flushed copper guard.

The pH is reported on the total hydrogen ion concentration scale (see *e.g.*, [84]). Calibrations for sensors 6–8 were carried out pre-deployment in the MBARI test tank, where tank pH was measured using the spectrophotometric method [85]. Vicarious calibration of sensors 3, 4, and 11–15 was accomplished by first deploying the sensor and then collecting one or more discrete samples within <1 m of the sensor, usually several days after deployment; sensors 2, 9, and 10 were calibrated in a common vessel prior to deployment with vicarious *in situ* calibration immediately following. Analyses of discrete samples used for vicarious calibration and subsequent calculation of pH at the deployment temperature were carried out as recommended by Dickson *et al.* [86]. Temperature dependence of the SeaFET sensor was accounted for as described in ref. [37]. Sensors 1 and 5 were calibrated to a point, within ∼1 week of mooring deployment, where pH was calculated from the pCO_2_ measured by the self-calibrating PMEL MapCO2 sensor co-located on the mooring and a total alkalinity value calculated from a region-specific conservative relationship between total alkalinity and salinity: A_T_ = 2131+50.8*(Salinity−31.25) (equation provided by Simone Alin).

As discussed in ref. [37], DuraFET sensors operate with short term precision of ±0.0005 pH units and exhibit stability over weeks to months of ±0.005 pH. In situ validation of sensor stability was not incorporated into every sensor deployment presented here, but in several cases (*e.g.* Palmyra, CCE1 mooring) ancillary data were available that allowed us to confirm this stability of the sensors. Several factors combine to determine the overall uncertainty of the sensor pH value; the 1st-order error is due to the uncertainty in the pH of the solution in contact with the sensor at the time that a pH value is ascribed as a calibration point. Because the bottle samples were processed using high accuracy CO_2_ analysis methods, we attribute the majority pH uncertainty to sampling error due, for example, to small scale gradients at the deployment site when an in situ sample is collected for vicarious calibration. It is therefore likely that the uncertainty of pH is highest at the most dynamic sites because these locations exhibit the most intense gradients. Yet establishing uncertainty in sampling error requires a constant field presence to carry out a meaningful number of discrete samples over an appropriate period of time. Such validation is beyond the scope of the studies presented in this work. Based on past in situ validations, we (KSJ, TRM) have observed sampling errors ranging from ±0.0007 to ±0.015 pH units. We therefore report here that the worst-case uncertainty in pH is around ±0.015, and in many instances, we expect the uncertainty to be better than ±0.01 pH units. In our experience, biofouling can compromise data quality for the passively flushed SeaFET within ∼2 months of deployment in high fouling environments, and, in general, the pumped version remains stable for much longer periods. In every case shown in Figure 1, the 30-day window was selected early in the time series to avoid deleterious effects of biofouling. We are now archiving the quality-controlled time series presented in this work at http://martzlab.ucsd.edu/data.html. Longer time series from CCE1 and CCE2 can be viewed at http://mooring.ucsd.edu/projects/cce/cce_data.html.

As noted in Table 1, data were recorded in the upper 15 m of the ocean. However, the water depth at each location was highly variable with some of the deployment locations categorized as pelagic and others benthic (*i.e.*, within 5 m of the bottom; Table 1).
